# Ranking stressor impacts on periphyton structure and function with mesocosm experiments and environmental-change forecasts

**DOI:** 10.1371/journal.pone.0204510

**Published:** 2018-09-24

**Authors:** David M. Costello, Konrad J. Kulacki, Mary E. McCarthy, Scott D. Tiegs, Bradley J. Cardinale

**Affiliations:** 1 School for Environment and Sustainability, University of Michigan, Ann Arbor, Michigan, United States of America; 2 Department of Biological Sciences, Kent State University, Kent, Ohio, United States of America; 3 Exponent, Maynard, Massachusetts, United States of America; 4 Department of Biological Sciences, Oakland University, Rochester, Michigan, United States of America; INRA, FRANCE

## Abstract

Streams are being subjected to physical, chemical, and biological stresses stemming from both natural and anthropogenic changes to the planet. In the face of limited time and resources, scientists, resource managers, and policy makers need ways to rank stressors and their impacts so that we can prioritize them from the most to least important (i.e., perform ‘ecological triage’). We report results from an experiment in which we established a periphyton community from the Huron River (Michigan, USA) in 84 experimental ‘flumes’ (stream mesocosms). We then dosed the flumes with gradients of six common stressors (increased temperature, taxa extinctions, sedimentation, nitrogen, phosphorus, and road salt) and monitored periphyton structure and function. A set of *a priori* deterministic functions were fit to each stressor–endpoint response and model averaging based on AICc weights was used to develop concentration–response best-fit predictions. Model predictions from different stressors were then compared to forecasts of future environmental change to rank stressors according to the potential magnitude of impacts. All of the stressors studied altered at least one characteristic of the periphyton; however, the extent (i.e., structural and functional changes) and magnitude of effects expected under future forecasts differed significantly among stressors. Elevated nitrogen concentrations are projected to have the greatest combined effect on stream periphyton structure and function. Extinction, sediment, and phosphorus all had similar but less substantial impact on the periphyton (e.g., affected only structure not function, smaller magnitude change). Elevated temperature and salt both had measurable effects on periphyton, but their overall impacts were much lower than any of the other stressors. For periphyton in the Huron River, our results suggest that, among the stressors examined, increased N pollution may have the greatest potential to alter the structure and function of the periphyton community, and managers should prioritize reducing anthropogenic sources of nitrogen. Our study demonstrates an experimental approach to ecological triage that can be used as an additional line of evidence to prioritize management decisions for specific ecosystems in the face of ecological change.

## Introduction

Earth’s ecosystems are increasingly being subjected to a long list of abotic and biotic forms of stress [1,2]. Eutrophication, climate change, biodiversity loss, and invasive species are just a few of the stressors that are altering the structure and functioning of ecosystems, and all have received international attention in policy and management (e.g., [2,3]). Inherent within this often-overwhelming list of stressors is the question: How can we identify which stressors have the greatest impacts on ecosystems and determine which deserve the highest priorities for research, policy, and management? Funding, time, and personnel are too limited to address each of the pressing environmental problems we face simultaneously. Given this, it is necessary to perform some type of ‘ecological triage’ in which stressors can be ranked by their relative impacts and prioritize accordingly (as advocated by [4–7] for their various disciplines).

A number of different methods have been used to rank the effects of different stressors on the structure and function of ecosystems. One of the more common approaches relies on expert opinion, in which individuals score the impacts of various forms of environmental change based on their personal knowledge and experience. This approach has been used to rank the impacts of stressors contributing to biodiversity loss (e.g., [8,9]), and to map the ‘health’ of various regions or ecosystems (e.g., [10,11,12]). While expert knowledge allows management decisions to progress even in the face of imperfect or incomplete knowledge, the risk of expert opinions is they are often qualitatively derived, inconsistent from expert to expert, and sometimes prove to be incorrect.

More quantitative methods for comparing stressors include field surveys and meta-analyses. Field surveys are widely used to correlate the magnitude of stressors with impacts on ecosystems (e.g., [10,13,14]). This approach benefits from maximal reality, but suffers from a common inability to unambiguously link cause to effect—that is, to show that the presumed stressor is indeed the agent of change in a response variable. A different approach was highlighted in the paper by Hooper and colleagues [15] in which the authors compared stressors by summarizing the results of many published meta-analyses to rank effect sizes of various aspects of environmental change on plant biomass production. This ‘meta-meta’ analysis (i.e., a meta-analysis of existing meta-analyses) included a massive number of studies providing what is perhaps the broadest possible inference about stressor impacts. But a limitation of such analyses is that they risk comparing apples-to-oranges, meaning they compare stressors that were measured or manipulated in very different ways, in different systems, using different organisms. This approach may not be useful for managers making local decisions about a particular ecosystem.

Another approach to ranking environmental stressors is the use of comparative experiments. Comparative experiments are common in fields such as ecotoxicology, where concentration–response curves are generated for numerous chemical stressors simultaneously and then used to rank the stressors from highest to lowest priority based on some quantitative metric such as the concentration that is lethal to 50% of an exposed population (LC50) [16]. The comparative experimental approach is a way of gaining stronger inference about the relative impacts that different stressors have on the structure and function of ecosystems [17]. However, because comparative experiments often require a large number of experimental treatments and replicates for sufficient statistical power, they are often limited to small spatial scales (test tube or bottle experiments), focus on a select group of model organisms, and lack many of the interactions among organisms that are present in the systems they intend to mimic.

All of these ecological triage approaches have limitations with regards to predicting effects of interacting stressors. Experts frequently struggle with non-additive stressor interactions, particularly when there exists no empirical data on interacting stressors [18]. The ambiguity in assigning causality from field surveys of impaired ecosystems may be a result of the cumulative and often interacting effects of multiple stressors on biological endpoints, but they are difficult to disentangle. Comparative experiments can systematically study stressor interactions, but experiments studying more than two or three stressor combinations are rare [19] likely due to logistical challenges. Although stressor interactions are an increasingly frequently target of study [19,20], as whole, we argue that it may be possible to ignore stressor interactions for initial ecological triage. If stressors interact additively, then information about how ecosystems respond to a stressor in isolation can predict multiple stressor effects. Furthermore, if stressors interact antagonistically then single stressor comparative experiments provide a conservative estimate of stressor effects under future scenarios of multiple stressors [20–22]. As a worst-case scenario, stressors that interact synergistically would cause predictions from single stressor comparative experiments to underestimate or perhaps completely misrank stressor importance. However, recent metaanalyses on multiple stressors demonstrate that synergistic stressor interactions are less common (i.e., 15–30% of studies) than additive and antagonistic interactions [19,20,23]. Therefore, single stressor comparative experiments may provide an excellent first step in ecological triage; these studies can identify the stressor most likely to cause impairment under additive and antagonistic interactions, and will provide guidance for future studies to identify potential synergism between the primary stressor and co-occurring stressors.

Here we report the results of a set of comparative experiments in which we manipulated the intensity of six common forms of ‘stress’ (nitrogen (N), phosphorus (P), salt, temperature, sediment, and taxon extinction) to quantify and rank their relative impacts on the structure and function of stream periphyton. These stressors were chosen for their broad regional and global importance [10,24], as well as their previously documented potential to impact stream structure and function [24,25]. We focused on periphyton due to its importance as a basal resource in stream food webs [26,27], a primary driver of many critical ecosystem functions [28,29], and its sensitivity to stress [30]. We inoculated stream mesocosms with water and periphyton from a focal river (Huron River, MI, USA) and applied the stressors of interest to isolate the potential effects of each stressor. We used a ‘concentration–response’ style analysis to quantify the magnitude of change in (1) periphyton community composition, (2) algal primary productivity, and (3) biofilm elemental content (carbon [C], N, and P) per unit change in each stressor. We then used multimodel inference and model averaging to produce quantitative estimates of stressor effects across a range of concentrations and directly compare these effects on each response variable under forecasted levels of stress. With the caveat that mesocosms are only a caricature of real streams, we argue that our study complements other forms of ecological triage that quantify and rank the impacts of various environmental stressors so that these vital ecosystems can be more effectively and efficiently managed.

## Materials and methods

### Study system

As a focal system, we chose the benthic community that inhabits the Huron River running through the University of Michigan’s Nichols Arboretum in Ann Arbor, MI (42°16′59.32″ N, 83°43′27.89″ W). At this location, the Huron River is a 5^th^ order stream that drains 1,955 km^2^ in southeast Michigan, USA with a mean annual discharge of 13.2 m^3^ sec^-1^ (USGS Station #04174500). On October 24, 2011, we used a submersible pump to collect 3000 liters of water from the river. Water was transported to the Cardinale lab experimental ‘flume facility’ on the central campus of the University of Michigan, where it was passed through a coarse screen (63-μm) and stored in two opaque holding tanks. One holding tank was used to fill the aquatic mesocosms on the next day (see below) and the second storage tank was used for weekly water exchanges in the mesocosms. Water stored for >24-h was recirculated through an ultraviolet sterilizer (Aqua Ultraviolet, USA).

### Stream mesocosms

**Fig 1:**
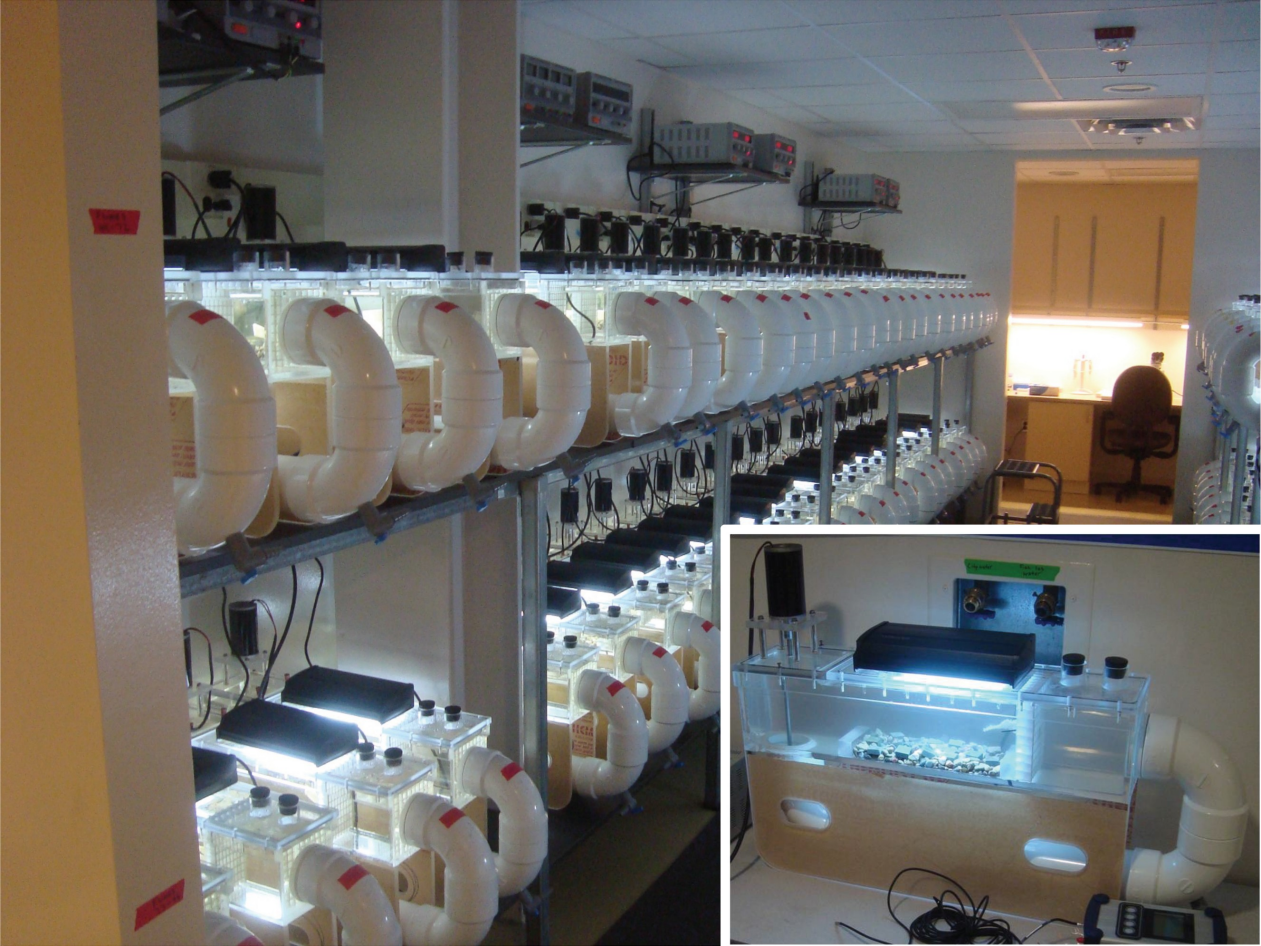
Images of the re-circulating mesocosms shown in use during the experiment. Inset: Close-up of one re-circulating flume including the DC motor at top left, fluorescent light on top, and pea gravel and tiles in the flume.

The Cardinale lab flume facility houses 144 recirculating mesocosms (hereafter, flumes), each of which is 0.6 m length × 0.1 m width × 0.1 m height and holds 13.3-L of water (Fig 1). A 7-cm diameter propeller controlled by a DC motor attached to a TechPower HY3020E 3-amp voltage regulator maintains water flow in each unit. Water velocity in each flume was held at a constant 20 cm sec^-1^ (SD = 0.02). To a 270-cm^2^ working section on the bottom of each flume, we added 600 mL of pea-sized gravel (0.5–2 cm diameter) and fifteen slate tiles (4 cm^2^) that served as substrates for algal colonization. The tiles provided a substrate with a standardized area to simplify sampling algae. Lighting was provided by Coralife Aqualight T5 light fixtures containing two 9-watt, 10K daylight spectrum fluorescent lamps, set to a 12:12-h light:dark cycle. Air temperature in the lab was maintained at 18.3 ± 1.1°C, resulting in ambient water temperatures of 19.8 ± 0.4°C.

On October 25, 2011, we collected 25 cobbles evenly spaced along transects in riffle and run habitats of the Huron River. We gently removed the biofilm from these rocks with a soft toothbrush into stream water, and took the biofilm slurry to the laboratory where it was passed through a 250-μm sieve to remove macroinvertebrates and large detritus. We homogenized the slurry in a blender, and subsamples were collected to determine cell densities. We then added 30 mL of slurry (equal to 2.0×10^6^ algal cells) to each flume with exception of flumes assigned to the extinction treatment, which received a modified inoculum. Seven days later (day 8 of the experiment), we repeated the biofilm collection and performed a second inoculation (1.7×10^6^ algal cells per flume) to ensure successful establishment. The algal slurry used to inoculate all but the extinction flumes contained 43 algal taxa. We used a 4-fold dilution series of the slurry to generate 6 levels of reduced taxa abundance (i.e., 1/4 to 1/4096 dilution of the slurry). Taxa richness in the serial dilutions were checked microscopically, which confirmed that each step of the dilution series had lower taxa richness (11–37 taxa in inoculum, Table 1). Rare taxa were eliminated from diluted samples, but taxa that were abundant in the undiluted inoculum (e.g., *Nitzschia*, *Navicula*, and *Limnothrix*) remained even in the most dilute inoculum.

**Table 1:** Summary of stressors manipulation including the magnitude of each of treatment.

Stressor	Manipulated as	Treatment levels^a^	Ambient condition	‘Large’ increase condition	Citations
Extinction	Diluted inoculum	14, 30, 35, 56, 67, 74% less taxa in inoculum	0% taxa loss	50% taxa loss	[15,32]
Nitrogen	NaNO_3_	547, 730, 912, 1094, 1459, 1824 μg NO_3_^-^-N L^-1^	365 μg N L^-1^	2.7-fold increase	[33]
Phosphorus	KH_2_PO_4_	51, 82, 114, 145, 208, 272 μg P L^-1^	19 μg P L^-1^	2.4-fold increase	[33]
Salt	NaCl	159, 318, 476, 635, 794, 953 mg Cl^-^ L^-1^	80 mg Cl^-^ L^-1^	330 mg Cl^-^ L^-1^	[34,35]
Sediment	Silt & clay	25, 50, 100, 200, 400, 800 mg TSS L^-1^	13 mg TSS L^-1^	284 mg TSS L^-1^	[34,36,37]
Temperature	Heaters	-0.5, +0.2, +0.4, +1.0, +1.2, +1.7, +1.9, +2.8, +3.2, +3.2, +3.8 °C	19.8 °C^b^	+4 °C	[38–40]

Ambient initial conditions in flumes approximated conditions in the Huron River, MI, USA with the exception temperature (see footnotes). Citations provide rationale for the selected range of stressor values and reference ‘large’ increases in stress.

^a^ Each treatment level (with the exception of temperature) was replicated twice

^b^ Mean daily water temperatures in the Huron River were 14–18°C in two weeks preceding our study [34]. Ambient flume temperatures were slightly greater due to heat generated by the artificial lights used on the mesocosms.

### Stressor treatments

Each of 72 flumes were assigned to one of six different stressor treatments that represent potential stressors to Great Lakes streams like the Huron River: species extinction, N, P, salt, sediment, and temperature (Table 1). An additional twelve flumes were assigned to serve as unmanipulated controls. Each stressor treatment had six treatment levels replicated twice (with the exception of temperature, which had 12 unreplicated treatments); we chose levels of each stressor to span a gradient from near-ambient conditions in the Huron River to high levels of stress that have been predicted to occur in streams in the Great Lakes region over the next 50 years (citations given in Table 1). Levels of stress were manipulated by adding solutions of concentrated chemicals (N, P, and salt), adding collected fine sediment (<63 μm) from the bed of the Huron River (sediment), inoculating with a diluted biofilm slurry (extinction), or applying heaters (temperature). All stressors other than extinction were imposed on the flumes after streams had reached a steady state biomass (Day 17–24; see S1 Fig. for full time-series). Our study was designed using a regression approach, which provides a statistically robust method to estimate functional relationships between individual stressors and periphyton response and allows for comparison among stressors [31].

### Periphyton sampling

Starting on Day 10 of the experiment, we collected weekly samples from the flumes for measurement of periphyton biomass. One randomly chosen sampling tile was removed from each flume and placed in 90% EtOH in a freezer to extract photosynthetic pigments (24 h). To estimate algal biomass we measured fluorescence of chlorophyll *a* on a Synergy H1 Hybrid microplate reader (BioTek, USA). On Day 15 of the experiment, we began weekly exchanges of 10% of the flume volume (1.33 L). Weekly 10% water changes were not sufficient to maintain nutrient concentrations and pH at in-stream initial conditions, and end of experiment nutrient concentrations declined to below detection limits and pH rose from 8.1 to 8.5 (see repository data). However larger volume or more frequent water replacement was not feasible for 72 flumes, and the trajectory of changes in water chemistry was similar among all flumes (with the exception of the intended nutrient treatments). Weekly water exchanges and algal biomass sampling continued for an additional six weeks (total experimental duration 56 days).

At the end of the experiment, after periphyton biomass had attained steady-state conditions for several weeks (S1 Fig), we measured primary production in the flumes using a ^13^C tracer-uptake study [41,42]. On days 49–50 of the experiment, we scrubbed tiles and collected biofilm onto glass fiber filters (0.7 μm pore size) to establish pre-addition concentrations of ^13^C in the periphyton. On Day 51, we performed alkalinity titrations to determine concentrations of dissolved inorganic carbon (DIC) concentrations in each flume [43]. Then, on days 55–56, we added 1.059 mmol NaH^13^CO_3_ to all flumes, which led to a trivial increase in DIC (2.6%), but a 330% increase in dissolved ^13^C. Lights were kept on for the duration of the isotope addition, which lasted approximately 15 h. On days 56–57, we again collected samples of periphyton on glass filters to determine post-addition concentrations of ^13^C in the biofilm. Pre- and post-addition isotope concentrations were measured on a Delta-Plus isotope ratio mass spectrometer at the University of Michigan’s isotope lab. Rates of primary production were calculated as:

$$Pn=\frac{C*(a_{post}-a_{pre})}{t*(a_{DIC}-a_{pre})}$$

where *Pn* is the photosynthetic rate (μg C cm^-2^ hr^-1^), *C* is the mass of organic carbon in the biofilm sample (μg C cm^-2^), *t* is the duration of the incubation (hr), and *a* terms are isotopic ratios of C (i.e., ^13^C/^12^C) in the periphyton post-addition (*a*_*post*_), periphyton pre-addition (*a*_*pre*_), and as DIC in the flume (*a*_*DIC*_) [41].

Also at the end of the experiment, we quantified algal community composition, and periphyton elemental content (C, N, and P concentrations). Using a soft brush, we scrubbed all biofilm from two slate tiles and fifteen haphazardly selected pieces of gravel from each flume into 100 mL of filtered Huron River water (from the storage tank). For community composition, we preserved a 40-mL subsample in 2% formalin for microscopic analysis. Samples were counted on an Olympus BX50 microscope at 400× magnification using a hemacytometer; all individuals were identified to genus. We then scaled cell counts to an areal basis (cm^-2^) by scanning the fifteen pieces of gravel and two tiles from each flume on a flatbed scanner, digitizing the image, and determining how much area had been scrubbed using Adobe Photoshop.

Periphyton C, N, and P concentrations were measured by filtering 20-mL aliquots through two pre-ashed, pre-weighed glass fiber filters (0.7 μm), which were subsequently dried at 60°C (>24 h). We analyzed one filter for total P by combusting the designated filter at 450 °C for four hours, then digesting it in 5 mL of 1M hydrochloric acid at 80°C (30 min) [44]. Digestate was diluted to 100 mL with Milli-Q water and analyzed for soluble reactive P using the ascorbic acid method (APHA 1999). The second filter was ground with a mortar and pestle, and 1–2 mg of algal material was measured into a tin capsule and analyzed for total C and total N on a Carlo Erba NC2500 elemental analyzer.

### Data analysis

A single flume (temperature treatment) was excluded from all analyses due to failure of the heater, and some individual samples were damaged and excluded from analysis. No more than 1 replicate per treatment was lost for any response variable (a single *Pn* measurement each from sediment, phosphorus, and control flumes and one elemental content sample from sediment). Algal community composition was compared among stressor treatments using canonical correspondence analysis (CCA) performed with the ‘vegan’ package within R 3.2.4 [45]. We selected CCA to analyze our algal community because it is a constrained ordination technique [45,46]; this experiment in replicated flumes allowed for tight control of environmental conditions, and our stressor treatments were likely to be the major driver of community structure. The matrix of algal taxa abundance was regressed against the linear combination of the stressors in absolute units (i.e., number of taxa lost from inoculum, water temperature, concentration of NO_3_^-^-N, PO_4_^3-^-P, Cl^-^, and TSS). Row and column scores were standardized by centering and normalizing prior to ordination, and we present a biplot of taxa scores for the first two CCA axes and vectors of the environmental variables [46]. We used a permutation test within ‘vegan’ to determine if (1) the full matrix of stressors, (2) individual constrained axes, or (3) specific stressors explained more variation in the community matrix than would be expected from random chance. Finally, for each taxon we calculated the proportion of total inertia that was explained by the significant constrained axes; those taxa with >10% inertia explained were deemed taxa significantly influenced by the stressors.

Comparing the impacts of multiple stressors required a model fitting procedure to make predictions about the functional response of periphyton for an individual stressor and a standardization procedure to compare among stressors measured on different scales with different units. To accomplish this, we first fit data to an *a priori* set of models that encompass a range of plausible concentration–response functions for continuous response variables: null, linear, quadratic, exponential, power, Monod, and two threshold functions (null left slope or null right slope). For each stressor–response pair, we calculated the sample-size-corrected Akaike Information Criteria (AICc) and weight of support (w_i_) for each model [47]. Commonly, there was similar support for multiple deterministic functions (i.e., Δ AICc ≤2), and therefore model averaging based on w_i_ was used to calculate a predicted concentration–response relationship. Models that did not explain sufficient variation in the periphyton response were excluded from the model averaging. Selection and exclusion of ‘poorly fit’ models was completed by giving all models that were no better than the null model (i.e., AICc < null model AICc) a weight of support equal to zero. All model fitting, selection, and model averaging was performed in R using a model selection and multimodel package (‘AICcmodavg’ 2.1–0), and threshold models were fit using the segmented regression package (‘segmented’ 0.2–9.2). Although we focus our paper on summarized results of the model selection exercise, detailed results from the model forms, fits, and weighting are reported in the Supplementary Information (S1–S4 Tables).

After determining the concentration–response relationship for each stressor–endpoint pair, we used these statistical relationships to predict which stressor would have the greatest potential to alter each response based on environmental projections of stress levels. Projected stress levels in the Huron River were estimated from environmental forecasts for 50 years in the future (see citations in Table 1). When forecasts were not available (i.e., salt and sediment), we identified watersheds of similar-size that have been highly impacted by the stressor, and used mean concentrations. Forecasts of future temperature is expected to vary spatially, and we used predictions specific to the Great Lakes region [38]. We determined the relative change in a given response variable by dividing the model-predicted estimate for the response at given exposure levels by the mean of the untreated controls at ambient conditions. For example, *Pn* was positively related to nitrate concentrations and a 2.7-fold increase in nitrate in our experiment increased rates of photosynthesis to 77.6 ng C cm^2^ h^-1^, representing a 34.3% increase in *Pn* over ambient rates (57.8 μg C cm^2^ h^-1^). By performing similar calculations for all stressors, we determined the relative change in the response variable expected under forecasted stress conditions (Table 1) and compared the magnitudes of change to each other. To summarize the potential impacts of the tested stressors across the periphyton endpoints, the stressors were ranked (greatest relative effect = 6, lowest relative effect = 1). Stressors were given a higher rank if they explained the most variation in response (i.e., community composition) or had the largest magnitude of effect at forecasted stress levels (i.e., *Pn* and elemental content). Stressors that did not cause a significant change in periphyton were given a rank of 0.

## Results

### Community composition

**Fig 2:**
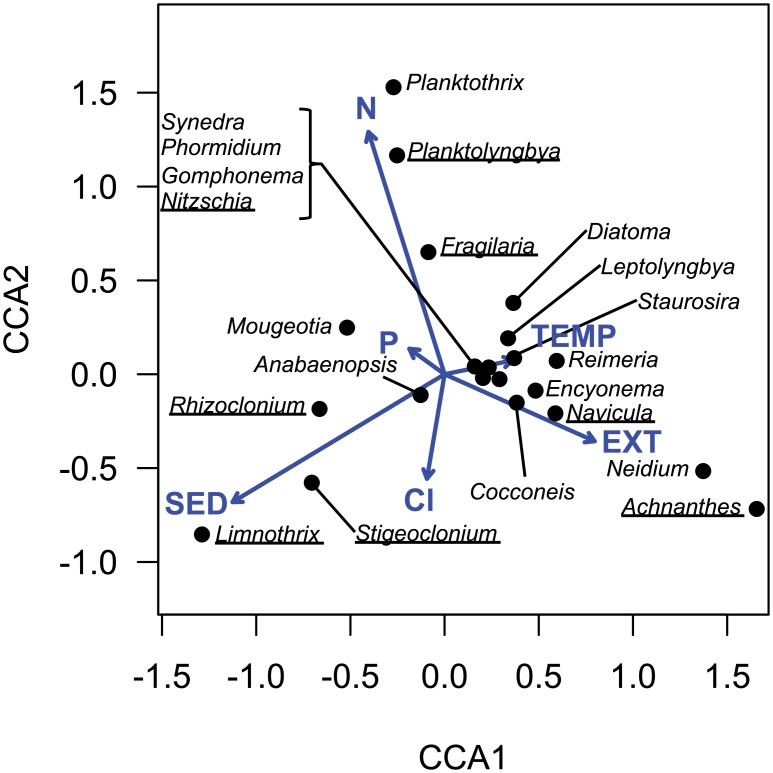
Canonical correspondence analysis (CCA) ordination biplot showing the relationships between abundance of algae genera found in mesocosms at the end of the experiment (points) and concentration of stressors (vectors). Taxa appropriately explained by the constrained ordination (i.e., >10% of inertia explained by axes 1–3) are underlined. Axis 1 explains 11.9% of variation in the community matrix, axis 2 explains 4.5%, and axis 3 (not shown) explains 3.0%. EXT = extinction, N = nitrogen, P = phosphorus, SED = sediment, Cl = salt, and TEMP = temperature.

Canonical correspondence analysis (CCA) indicated that the stressor treatments imposed a significant amount of variation in the algal community abundance matrix (permutation test, *P* = 0.001). Three constrained axes explained a greater amount of variation than expected by random chance; the first axis explained 11.9% of the community variation (*P* < 0.001), the second axis explained 4.5% of variation (*P* = 0.002), and the third axis explained 3.0% of variation (*P* = 0.01). Given that our experimental design included no interactions among stressors, it was not surprising that three stressors significantly structured the algal community and each of these stressors was correlated to a single axis. Sediment explained the greatest amount of community variation (*P* = 0.002) and was correlated to CCA axis 1. Nitrogen was correlated to CCA axis 2 (*P* = 0.017), and the extinction treatment was correlated to CCA axis 3 (*P* = 0.038). Overall, 8 of the 22 taxa present in the flumes at the end of the experiment had abundances that were significantly correlated (i.e., >10% inertia explained by CCA 1–3) to at least one stressor (Fig 2). Greater concentrations of sediment were associated with higher abundances of filamentous algae *Limnothrix*, *Stigeoclonium*, and *Rhizoclonium* and lower abundances of the diatom *Navicula* (Fig 2). Elevated concentrations of N were correlated with greater abundance of the diatom *Fragilaria* and cyanobacterium *Planktolyngbya* and lower abundance of *Stigeoclonium* (Fig 2). Although *Planktothrix* had high loadings for CCA axis 2, its abundance was highly variable (only present in two flumes) and was not significantly correlated to N concentrations. Flumes inoculated with the species-poor communities (i.e., high extinction) contained greater relative abundances of *Achnanthes* but lower abundances of *Nitzschia*. While extinction is expected to alter community structure, it is noteworthy that this stressor was primarily associated with increased abundance of taxa that were rare or absent from control flumes (most notably *Achnanthes*, but to a lesser extent *Neidium*, Fig 2). Phosphorus, salt, and temperature all had comparably weak effects (all *P* > 0.50), and were not clearly associated with shifts in the abundance of any specific taxa of algae.

### Primary production

**Fig 3:**
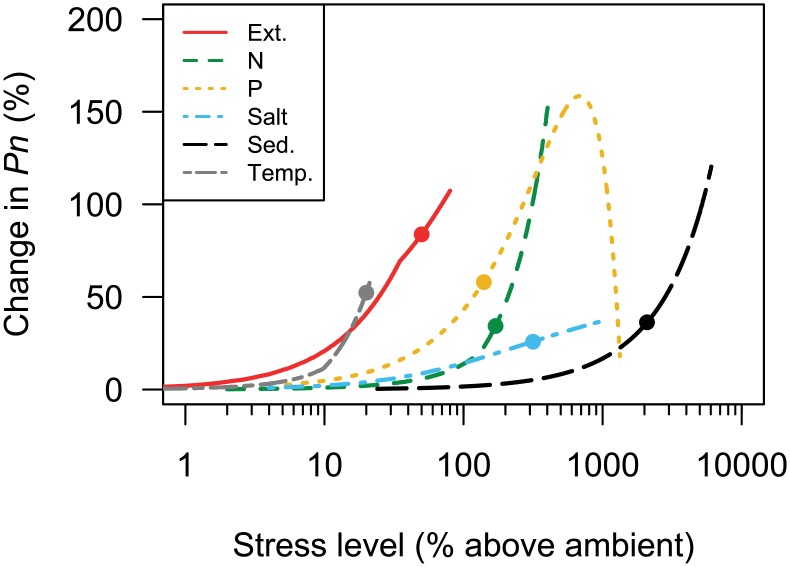
Best-fit model predictions (model averaging based in AICc weights) of the relationship between stressors and photosynthetic rate (*Pn*) expressed as a percent difference from ambient (stressors) or control streams (*Pn*). Each line represents a different stressor, and the length of each line represents the interpolated range of experimental treatments (Table 1). Symbols on each line indicate the predicted change in *Pn* given an increase in a single stressor under forecasted conditions (Table 1).

Elevated levels of stress increased rates of photosynthesis for all stressors. However, the magnitude of effects and functional fit differed among stressors, which indicates that the stressors had qualitatively different impacts on *Pn* (Table 2, Fig 3). Flumes treated with P increased *Pn* at low levels of stress but *Pn* declined when P concentrations exceeded 590 μg L^-1^. Nitrogen, extinction, sediment, salt, and temperature all increased *Pn* monotonically (Fig 3), but nitrogen, sediment, and temperature stress exhibited more of a threshold concentration–response relationship when compared to the relatively gradual response of *Pn* to increased extinction and salt (Fig 3, S1 Table). Under our forecasted stress scenarios (Table 1), we found that extinction had the largest potential effect on *Pn*, followed by elevated P, elevated temperature, elevated nitrogen, and finally salt (Table 2, Fig 3). Our data indicate that forecasted increases in extinction (+82%), elevated P (+58%), and elevated temperature (+52%) are predicted to cause substantial increases in *Pn* (Table 2, Fig 3). Forecasted changes in nitrogen, sedimentation, and salt are also predicted to increase *Pn*, but to a lesser amount than large changes in P, extinction, and temperature (Table 2).

**Table 2:** Summary of model averaged (based on AICc weights) periphyton response in photosynthetic rate and periphyton elemental content (C, N, and P) for forecasted increases in stress.

Stressor	Predicted change under forecast conditions (%)			
	Photosynthetic rate	Carbon	Nitrogen	Phosphorus
Extinction	83.8	15.2	0	0
Nitrogen	34.3	14.0	31.1	-20.4
Phosphorus	58.0	0	7.2	33.5
Salt	25.8	0	5.8	24.8
Sediment	36.3	-4.3	0	0
Temperature	52.3	78.0	0	0

Magnitude of forecasted increases in stress are detailed in Table 1.

### Periphyton elemental content

**Fig 4:**
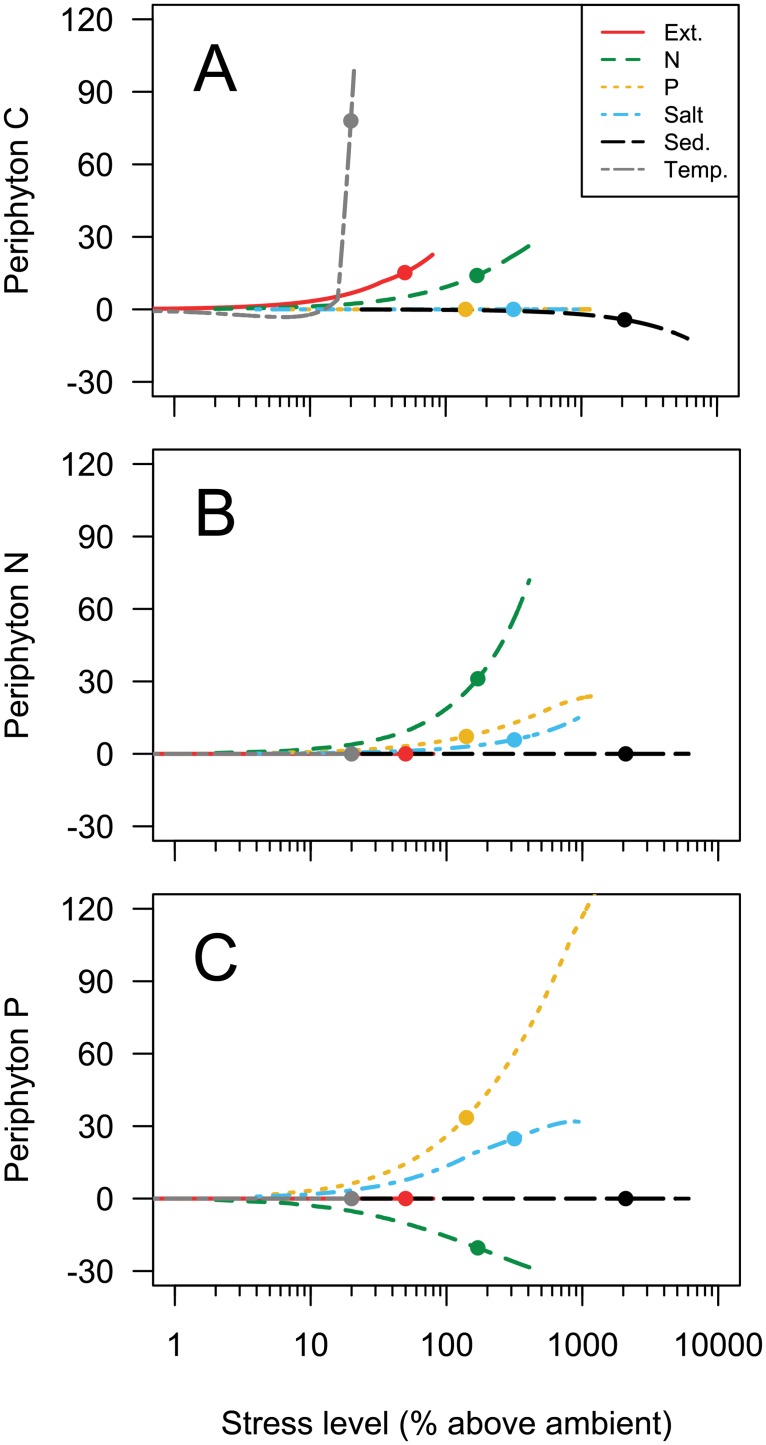
Best-fit model predictions (model averaging based in AICc weights) of the relationship between stressors and periphyton carbon (A), nitrogen (B), and phosphorus (C) concentrations expressed as a percent difference from ambient (stressors) or control streams (elemental content). Each line represents a different stressor, and the length of each line represents the interpolated range of experimental treatments (Table 1). Symbols on each line indicate the predicted change in *Pn* given an increase in a single stressor under forecasted conditions (Table 1).

The stressors studied in this experiment altered the elemental content of periphyton. In many cases, the periphyton chemistry response differed from the photosynthesis response and was inconsistent among elements. Similar to the periphyton production response, carbon content was positively related to temperature, extinction, and N concentrations (Fig 4). However, while elevated P had a strong stimulatory effect on *Pn*, it did not change periphyton carbon content (Table 2). Sediment caused a slight decline in periphyton C content at very high sediment concentrations, and salt did not significantly alter periphyton C (Fig 4A). Comparing among stressors, temperature treatments led to the greatest impact on periphyton carbon concentrations, ranking highest among stressors at forecasted levels of stress (Fig 4A; Table 2). Not surprisingly, elevated inorganic nutrient concentrations were associated with the greatest changes in periphyton N and P content (Fig 4, Table 2). Periphyton N concentrations increased in the presence of inorganic N, inorganic P, and salt but did not change significantly in the presence of any other stressors (Fig 4B, Table 2). Periphyton P content increased most in the presence of inorganic P, but increased salt concentrations also led to a substantial increased in cellular P (Fig 4C). Elevated streamwater N concentrations caused a decline in periphyton P content (Fig 4C, Table 2). Neither extinction, sediment, or temperature stress had any significant effect on the N and P content of periphyton (Fig 4).

## Discussion

Here we have reported the results of a laboratory mesocosm experiment in which we compared the effects of six common stressors on the structure, function, and community composition of stream periphyton from the Huron River in Michigan, USA. Our study is one of the first to combine comparative laboratory experiments and forecasts of future environmental conditions to quantitatively rank the effects of different stressors on periphyton structure and function. While laboratory experiments have inherent limitations (discussed below) our study details an approach of how we can perform ‘ecological triage’ by ranking the potential impacts of stressors on the structure and function of communities to prioritize stressors that pose the greatest risk of ecological change.

Not surprisingly, all of the stressors impacted at least one of our measures of periphyton structure and function, but the consistency of response across periphyton characteristics (i.e, community structure, productivity, elemental content) and the magnitude of response relative to other stressors allow us to complete our ranking of stressors. We identified N as the most important stressor of the Huron River periphyton among those we examined; this stressor had a broad and strong effect on periphyton relative to others (Table 3). Nitrogen at forecasted concentrations is predicted to modify community structure (2^nd^ strongest effect of 6 stressors), moderately increase periphyton productivity (5^th^ rank), and strongly alter periphyton elemental content (1–3^rd^ rank). As observed here, elevated inorganic N in streams has frequently been shown to alter periphyton structure and function [48,49]. The strong response of periphyton to elevated N concentrations was somewhat surprising given the relatively high N:P ratio of the Huron River water (NO_3_^-^-N:SRP (molar) = 42.5, Table 1). However, previous studies have demonstrated periphyton structural and functional response to increases in inorganic N in streams with high ambient N:P [49].

**Table 3:** Summary of stressor ranks under forecasted conditions (Table 1) for three major characteristics of periphyton.

Stressor	Community	Production	Chemistry^a^	Final score
Nitrogen	5	2	4.7	13.7
Extinction	4	6	1.7	11.7
Sediment	6	3	1.0	10.0
Phosphorus	0	5	3.7	8.7
Temperature	0	4	2.0	6.0
Salt	0	1	3.0	4.0

Under forecasted conditions, stressors predicted to affect a periphyton characteristic are given a ranking ≥1 and stressors with no effect on a periphyton characteristic were given a ranking of 0. Rank-order of stressors were determined by the magnitude of response with the stressor eliciting the largest change in a periphyton characteristic given a rank of 6 (second strongest magnitude change given rank 5, etc.). The final score is the sum of all ranks across the three measures of periphyton.

^a^ Ranks for chemistry are mean ranks for C, N, and P (Table 2).

Extinction was the second highest ranked stressor with potential impacts under future scenarios but this stressor did not affect as many periphyton characteristics as elevated N (Table 3). High levels of extinction altered the community structure (3^rd^ rank) and productivity (1^st^ rank) but had minimal effects on periphyton elemental content (i.e., moderate effects on C only). It is unsurprising that the extinction treatment influenced community structure, but it was somewhat surprising that sediment and N had stronger effects on community structure than extinction. Although our diluted inoculum provided a limited species pool to colonize the flumes, at steady-state the extinction flumes had no fewer (and no more) taxa than control flumes (mean taxa richness 7.9 and 7.7, respectively). We did see large differences in community structure between control and extinction flumes, and many taxa that were rare in the control flumes were abundant in the extinction flumes and vice versa. The cyanobacterium *Anabaenopsis* and diatoms *Diatoma* and *Encyonema* were present in the control flumes but not in the extinction flumes whereas the opposite was observed for the taxa *Planktolyngbya*, *Fragilaria*, and *Achnanthes*. This shift in community structure related to a dilute species inoculation corresponded to a periphyton community that was more productive. Although extinction did not directly alter ecosystem function through reduced species richness, extinction did indirectly alter ecosystem function by modifying community composition. Our results provide additional evidence for the potential large influence of community structure on ecosystem functioning [15, 50,51] and support theoretical predictions that ordered sequences of extinction, such as those ordered by rarity, should have very different consequences for the functioning of ecosystems than what is predicted by random assembly experiments [52].

Elevated concentrations of sediment and P ranked third and fourth with respect to cumulative stressor impacts (Table 3). High levels of sediment were predicted to have large changes in periphyton community structure (1^st^ rank), moderate changes in periphyton productivity, and minimal changes in elemental content (C only). The mechanism for increased productivity under high-sediment conditions may be linked to the community shift to a greater abundance of filamentous algal taxa. A greater abundance of filamentous algae in response to elevated sediment is likely due to the physical disturbance of the added sediment (i.e., burial of adnate diatoms). Filamentous forms of algae are often viewed as a nuisance but, as supported by our data, can often have similar or greater rates of productivity when compared to periphytic communities that are not dominated by filamentous forms [53]. High concentrations of P were predicted to have large effects on periphyton productivity (2^nd^ rank), P content (1^st^ rank), and N content (2^nd^ rank) but no significant effect on community structure or periphyton C content. Periphyton responses to elevated P were similar to responses to elevated N, with both nutrients inducing large changes in productivity and periphyton nutrient content. This is not surprising given the strong role nutrients play in structuring primary producers in streams [48,54]. Furthermore, these comparative experiments suggest that the periphyton community in the Huron River is co-limited by N and P because addition of either nutrient elicited a response of greater production [55,56]. Although co-limitation of periphyton by N and P is not uncommon (see reviews by [56,57]), this co-limitation has consequences for nutrient management in the Huron River and may inform future studies that explore multiple stressors (see below).

Finally, we placed salt and temperature stress into a lower tier of stressors where impacts are not likely to be severe for Huron River periphyton (Table 3). Our data indicate that elevated temperatures expected under future climate change scenarios may increase periphyton productivity and carbon content but have minimal effect on community composition and N and P content. Although we do not view elevated temperature as a critical stressor for ecological triage of the Huron River, the feedback between elevated CO_2_ expected under future climate scenarios and our observed increased periphyton C content and *Pn* is worthy of future study. Elevated salinity only had effects on *Pn* and N and P content, and the magnitude of change in Pn and elemental content in response to high salt was minimal in comparison to other stressors. Large increases in salinity in streams associated with increased impervious surface [35] are not predicted to have strong direct effects on Huron River periphyton. However, we caution that macroinvertebrates are known to be highly sensitive to elevated salinity [58] and our observation of altered periphyton nutrient content under high salt conditions may indicate a mechanism by which elevated salt indirectly affects benthic grazers. Given that benthic grazers are important top-down controls on periphyton biomass [59], this potential indirect effect requires further study before completely discounting elevated salt as a stressor. It is critical to note that this exercise is for direct effects on the periphyton community only and examination of other trophic levels may yield different results.

In addition to our limited scope of a single trophic level, there are additional limitations to this study that need to be kept in mind when interpreting results. First, our study was performed in stream mesocosms that are, by design, oversimplifications of the physical, chemical, and biological complexities of stream ecosystems. The utility of mesocosms lie in their ability to allow well-replicated, highly controlled studies that can unambiguously rank the *potential* effects of stressors on aquatic environments in the absence of confounding variables. However, results should be viewed solely as predictions until confirmation (or refutation) can be provided by complementary field-based experiments or surveys. Second, the six stressors we studied rarely occur in isolation, and there is always the potential of non-additive interactions among stressors that can exacerbate or ameliorate the individual effects documented here. While consideration of multi-stressor interactions can generate intractable experimental designs (for 6 stressors there are 63 possible treatment combinations), a fruitful avenue of future work might be to take the highest-ranking individual stressors and study their interactions. For example, given that we know that elevated N-alone is likely to have the strongest direct effects on periphyton, it would be fruitful to look at interactions between N and other stressors (e.g., extinction and P). Alternatively, if a particular stressor combination is expected and of interest to resource managers (e.g., elevated temperature and salt), this study would provide key baseline information to establish strong hypotheses for future multi-stressor experiments. Thus, the triage approach we demonstrate here both gives a conservative estimate of the top ranking stressor and also can be useful for informing future experiments of greater complexity.

With the caveats above in mind, we believe the approach used in this study is a useful approach for comparing the effects of different stressors on community structure and function. Pairing comparative experiments quantitatively with forecasts of future conditions under environmental change provides a defensible way of ranking the impacts of distinctly different stressors. We anticipate that these predictions will be accurate under conditions where these stressors found as mixtures interact additively or antagonistically, which current data suggest represent are the primary ways that these stressors will interact [19,20,23]. The methods we have developed can be adjusted for different baseline values, different benchmarks of interest, other stressors, and other ecosystems. Comparative experiments also have potential to complement other forms of ecological triage that attempt to rank the importance of various stressors using expert opinion (e.g., [8,9]), correlative field surveys and case studies (e.g., [10,13]), or meta-analyses that synthesize data from distinctly different systems (e.g., [15]). As such, the approach detailed here holds promise for helping resource managers objectively decide where to best appropriate their limited time, funding and personnel.

## Supporting information

S1 Appendix Description of candidate *a priori* deterministic functions. (PDF)

S1 Table Results of AIC multimodel inference and model weighting for stressor effects on periphyton photosynthetic rate. Models that did not explain sufficient variation in the periphyton response (i.e., AICc < null model AICc) were given a weight of 0 and not included in the model averaging (these models are highlighted in grey). Significance of the models (i.e. p-values) were determine by comparison to the null model with a likelihood ratio test (LRT). Model fits are reported as multiple R^2^ for linear models (linear, quadratic) and a quasi-R^2^ for non-linear models (squared correlation coefficient of predicted vs. observed Y).
(PDF)

S2 Table Results of AIC multimodel inference and model weighting for stressor effects on periphyton carbon content. Models that did not explain sufficient variation in the periphyton response (i.e., AICc < null model AICc) were given a weight of 0 and not included in the model averaging (these models are highlighted in grey). Significance of the models (i.e. p-values) were determine by comparison to the null model with a likelihood ratio test (LRT). Model fits are reported as multiple R^2^ for linear models (linear, quadratic) and a quasi-R^2^ for non-linear models (squared correlation coefficient of predicted vs. observed Y).
(PDF)

S3 Table Results of AIC multimodel inference and model weighting for stressor effects on periphyton nitrogen content. Models that did not explain sufficient variation in the periphyton response (i.e., AICc < null model AICc) were given a weight of 0 and not included in the model averaging (these models are highlighted in grey). Significance of the models (i.e. p-values) were determine by comparison to the null model with a likelihood ratio test (LRT). Model fits are reported as multiple R^2^ for linear models (linear, quadratic) and a quasi-R^2^ for non-linear models (squared correlation coefficient of predicted vs. observed Y).
(PDF)

S4 Table Results of AIC multimodel inference and model weighting for stressor effects on phosphorus content. Models that did not explain sufficient variation in the periphyton response (i.e., AICc < null model AICc) were given a weight of 0 and not included in the model averaging (these models are highlighted in grey). Significance of the models (i.e. p-values) were determine by comparison to the null model with a likelihood ratio test (LRT). Model fits are reported as multiple R^2^ for linear models (linear, quadratic) and a quasi-R^2^ for non-linear models (squared correlation coefficient of predicted vs. observed Y).
(PDF)

S1 Fig Periphyton growth expressed as fluorescence of chlorophyll *a* over time in the experiment. Each panel represents a different stressor and the separate lines correspond to the six increasing levels of each stressor listed in Table 1 (i.e., Level 6 is highest treatment). Data are means ± SE of the two replicates with the exception of temperature, which had eleven unreplicated treatment levels. Ext. = extinction, N = nitrogen, Sed. = sediment, P = phosphorus, and Temp. = temperature.
(PDF)
