# Supplementary material for: Ranking stressor impacts on periphyton structure and function with mesocosm experiments and environmental-change forecasts
Source: PLoS One. 2018 Sep 24;13(9):e0204510. doi: 10.1371/journal.pone.0204510 (PMC6152968; doi:10.1371/journal.pone.0204510)
Supplement: S1 Appendix — (PDF) [file pone.0204510.s001.pdf]

Appendix S1. Description of candidate *a priori* deterministic functions

To determine concentration–response relationships between each stressor (X) and response (Y), we used the following candidate deterministic functions:

Null: $Y = a$

Linear: $Y = bX + a$

Quadratic: $Y = b_1X + b_2X^2 + a$

Exponential: $Y = ae^{bX}$

Power: $Y = aX^b$

Monod: $Y = ((a*X)/(b-X))$

Threshold (null left slope): if $X <$ the predicted breakpoint, then $Y = a$
if $X \geq$ the predicted breakpoint, then $Y = b(X – \text{breakpoint}) – a$

Threshold (null right slope): if $X <$ breakpoint: $Y = bX + a$
if $X >$ breakpoint: $Y = b*\text{breakpoint} + a$
