# Supplementary material for: Ranking stressor impacts on periphyton structure and function with mesocosm experiments and environmental-change forecasts
Source: PLoS One. 2018 Sep 24;13(9):e0204510. doi: 10.1371/journal.pone.0204510 (PMC6152968; doi:10.1371/journal.pone.0204510)
Supplement: S1 Table — Models that did not explain sufficient variation in the periphyton response (i.e., AICc < null model AICc) were given a weight of 0 and not included in the model averaging (these models are highlighted in grey). Significance of the models (i.e. p-values) were determine by comparison to the null model with a likelihood ratio test (LRT). Model fits are reported as multiple R2 for linear models (linear, quadratic) and a quasi-R2 for non-linear models (squared correlation coefficient of predicted vs. observed Y). (PDF) [file pone.0204510.s002.pdf]

Table S1. Results of AIC multimodel inference and model weighting for stressor effects on periphyton photosynthetic rate. Models that did not explain sufficient variation in the periphyton response (i.e., AICc < null model AICc) were given a weight of 0 and not included in the model averaging (these models are highlighted in grey). Significance of the models (i.e. p-values) were determine by comparison to the null model with a likelihood ratio test (LRT). Model fits are reported as multiple R^2 for linear models (linear, quadratic) and a quasi-R^2 for non-linear models (squared correlation coefficient of predicted vs. observed Y).

**Extinction**	k[a]	AICc	ΔAICc	w_i	p-value	R^2
Linear	3	-89.41	0.00	0.32	< 0.001	0.48
Null right slope	4	-88.90	0.51	0.25	< 0.001	0.53
Exponential	3	-88.23	1.18	0.18	< 0.001	0.45
Quadratic	4	-88.18	1.23	0.17	< 0.001	0.52
Null left slope	4	-86.45	2.96	0.07	0.001	0.48
Null	2	-76.96	12.45	< 0.01		
Monod	3	-62.70	26.72	0	1.00	0.51
Power	3	-62.62	26.79	0	1.00	0.50

**Salt**	k	AICc	ΔAICc	w_i	p-value	R^2
Monod	3	-84.18	0.00	0.53	0.04	0.19
Power	3	-82.88	1.30	0.28	0.08	0.14
Null	2	-82.14	2.04	0.19		
Quadratic	4	-81.90	2.28	0	0.10	0.21
Linear	3	-81.47	2.71	0	0.18	0.08
Exponential	3	-81.27	2.91	0	0.23	0.08
Null left slope	4	-78.25	5.93	0	0.44	0.07
Null right slope	NA[b]					

**Phosphorus**	k	AICc	ΔAICc	w_i	p-value	R^2
Quadratic	4	-82.25	0.00	0.98	< 0.001	0.61
Monod	4	-73.01	9.24	0.01	0.01	0.36
Null right slope	3	-71.65	10.60	< 0.01	0.03	0.26
Power	3	-70.95	11.29	< 0.01	0.05	0.20
Linear	3	-67.94	14.31	< 0.01	0.09	0.13
Null	2	-67.48	14.77	< 0.01		
Exponential	3	-67.21	15.04	0	0.19	0.11
Null left slope	4	-64.87	17.38	0	0.26	0.12

**Sediment**	k	AICc	ΔAICc	w_i	p-value	R^2
Exponential	3	-98.76	0.00	0.41	< 0.001	0.54
Linear	3	-98.40	0.36	0.34	< 0.001	0.54
Quadratic	4	-95.71	3.04	0.09	< 0.001	0.54
Null left slope	4	-95.39	3.36	0.08	< 0.001	0.54
Null right slope	4	-95.35	3.40	0.08	< 0.001	0.54
Null	2	-84.17	14.59	< 0.01		
Power	3	-61.16	37.60	0	1	0.37
Monod	3	-60.45	38.30	0	1	0.32

**Nitrogen**	k	AICc	ΔAICc	w_i	p-value	R^2
Exponential	3	-105.60	0.00	0.34	< 0.001	0.64
Quadratic	4	-105.34	0.26	0.30	< 0.001	0.68
Null left slope	4	-105.05	0.55	0.26	< 0.001	0.68
Linear	3	-102.58	3.02	0.07	< 0.001	0.59
Power	3	-99.78	5.82	0.02	< 0.001	0.54
Null right slope	4	-99.39	6.21	0.01	< 0.001	0.58
Monod	3	-96.77	8.83	< 0.01	< 0.001	0.48
Null	2	-84.78	20.82	< 0.01		

**Temperature**	k	AICc	ΔAICc	w_i	p-value	R^2
Null left slope	4	-100.21	0.00	0.21	0.05	0.26
Exponential	3	-99.96	0.25	0.19	0.08	0.15
Power	3	-99.82	0.39	0.17	0.09	0.14
Linear	3	-99.67	0.54	0.16	0.09	0.13
Quadratic	4	-99.35	0.87	0.14	0.08	0.23
Null	2	-99.20	1.01	0.13		
Null right slope	NA					
Monod	NA					

[a] Number of model parameters (including error), [b] function unable to fit the data
