# Supplementary material for: Ranking stressor impacts on periphyton structure and function with mesocosm experiments and environmental-change forecasts
Source: PLoS One. 2018 Sep 24;13(9):e0204510. doi: 10.1371/journal.pone.0204510 (PMC6152968; doi:10.1371/journal.pone.0204510)
Supplement: S2 Table — Models that did not explain sufficient variation in the periphyton response (i.e., AICc < null model AICc) were given a weight of 0 and not included in the model averaging (these models are highlighted in grey). Significance of the models (i.e. p-values) were determine by comparison to the null model with a likelihood ratio test (LRT). Model fits are reported as multiple R2 for linear models (linear, quadratic) and a quasi-R2 for non-linear models (squared correlation coefficient of predicted vs. observed Y). (PDF) [file pone.0204510.s003.pdf]

Table S2. Results of AIC multimodel inference and model weighting for stressor effects on periphyton carbon content. Models that did not explain sufficient variation in the periphyton response (i.e., AICc < null model AICc) were given a weight of 0 and not included in the model averaging (these models are highlighted in grey). Significance of the models (i.e. p-values) were determine by comparison to the null model with a likelihood ratio test (LRT). Model fits are reported as multiple R^2 for linear models (linear, quadratic) and a quasi-R^2 for non-linear models (squared correlation coefficient of predicted vs. observed Y).

**Extinction**	k[a]	AICc	ΔAICc	w_i	p-value	R^2
Linear	3	136.09	0.00	1.00	0.04	0.17
Exponential	3	136.15	0.06	0	0.05	0.17
Null	2	137.95	1.86	0		
Null right slope	4	137.98	1.89	0	0.09	0.20
Quadratic	4	138.80	2.71	0	0.13	0.18
Null left slope	4	138.99	2.90	0	0.14	0.17
Power	3	196.78	60.69	0	1.00	0.14
Monod	NA[b]					

**Salt**	k	AICc	ΔAICc	w_i	p-value	R^2
Null	2	122.60	0.00	1.00		
Power	3	125.21	2.61	0	0.90	< 0.01
Exponential	3	125.22	2.61	0	0.91	< 0.01
Linear	3	125.22	2.61	0	0.91	< 0.01
Quadratic	4	127.56	4.95	0	0.78	0.02
Null right slope	4	127.91	5.31	0	0.91	0.01
Null left slope	4	127.93	5.32	0	0.91	0.01
Monod	4	128.06	5.46	0	1.00	< 0.01

**Phosphorus**	k	AICc	ΔAICc	w_i	p-value	R^2
Null	2	122.23	0.00	1.00		
Quadratic	4	123.84	1.62	0	0.18	0.15
Linear	3	124.70	2.48	0	0.72	0.01
Exponential	3	124.71	2.49	0	0.72	0.01
Monod	3	124.85	2.62	0	0.93	< 0.01
Power	3	124.86	2.62	0	0.94	< 0.01
Null right slope	4	127.60	5.37	0	0.93	0.01
Null left slope	NA					

**Sediment**	k	AICc	ΔAICc	w_i	p-value	R^2
Exponential	3	113.28	0.00	0.35	0.11	0.12
Linear	3	113.36	0.08	0.33	0.12	0.11
Null	2	113.44	0.16	0.32		
Null right slope	4	114.36	1.08	0	0.13	0.19
Quadratic	4	115.56	2.28	0	0.22	0.14
Null left slope	4	116.36	3.07	0	0.31	0.11
Power	NA				1.00	0.14
Monod	NA					

**Nitrogen**	k	AICc	ΔAICc	w_i	p-value	R^2
Power	3	119.04	0.00	0.23	0.01	0.29
Monod	3	119.37	0.33	0.20	0.01	0.28
Linear	3	119.42	0.39	0.19	0.01	0.28
Exponential	3	119.61	0.57	0.17	0.01	0.27
Null right slope	4	120.95	1.91	0.09	0.02	0.32
Quadratic	4	121.64	2.61	0.06	0.02	0.30
Null left slope	4	122.36	3.32	0.04	0.03	0.28
Null	2	124.67	5.63	0.01		

**Temperature**	k	AICc	ΔAICc	w_i	p-value	R^2
Null left slope	4	122.66	0.00	0.77	0.003	0.43
Quadratic	4	125.29	2.62	0.21	0.01	0.36
Null	2	129.88	7.22	0.02		
Exponential	3	129.92	7.25	0	0.13	0.11
Linear	3	130.14	7.47	0	0.14	0.10
Null right slope	NA					
Power	NA					
Monod	NA					

[a] Number of model parameters (including error), [b] function unable to fit the data
