# Supplementary material for: Ranking stressor impacts on periphyton structure and function with mesocosm experiments and environmental-change forecasts
Source: PLoS One. 2018 Sep 24;13(9):e0204510. doi: 10.1371/journal.pone.0204510 (PMC6152968; doi:10.1371/journal.pone.0204510)
Supplement: S3 Table — Models that did not explain sufficient variation in the periphyton response (i.e., AICc < null model AICc) were given a weight of 0 and not included in the model averaging (these models are highlighted in grey). Significance of the models (i.e. p-values) were determine by comparison to the null model with a likelihood ratio test (LRT). Model fits are reported as multiple R2 for linear models (linear, quadratic) and a quasi-R2 for non-linear models (squared correlation coefficient of predicted vs. observed Y). (PDF) [file pone.0204510.s004.pdf]

Table S3. Results of AIC multimodel inference and model weighting for stressor effects on periphyton nitrogen content. Models that did not explain sufficient variation in the periphyton response (i.e., AICc < null model AICc) were given a weight of 0 and not included in the model averaging (these models are highlighted in grey). Significance of the models (i.e. p-values) were determine by comparison to the null model with a likelihood ratio test (LRT). Model fits are reported as multiple R^2 for linear models (linear, quadratic) and a quasi-R^2 for non-linear models (squared correlation coefficient of predicted vs. observed Y).

**Extinction**	k[a]	AICc	ΔAICc	w_i	p-value	R^2
Null	2	-5.00	0.00	1.00		
Exponential	3	-2.70	2.30	0	0.59	0.01
Linear	3	-2.69	2.31	0	0.59	0.01
Quadratic	4	-0.51	4.49	0	0.63	0.04
Null right slope	4	0.23	5.24	0	0.88	0.01
Monod	3	49.45	54.45	0	1.00	< 0.01
Power	3	49.50	54.50	0	1.00	< 0.01
Null left slope	NA[b]					

**Salt**	k	AICc	ΔAICc	w_i	p-value	R^2
Exponential	3	-11.00	0.00	0.28	0.09	0.13
Linear	3	-10.97	0.04	0.28	0.09	0.12
Power	3	-10.54	0.47	0.23	0.12	0.11
Null	2	-10.41	0.59	0.21		
Monod	3	-10.06	0.94	0	0.15	0.09
Quadratic	4	-8.22	2.78	0	0.23	0.13
Null left slope	4	-8.10	2.90	0	0.24	0.13
Null right slope	4	-8.04	2.97	0	0.25	0.12

**Phosphorus**	k	AICc	ΔAICc	w_i	p-value	R^2
Power	3	-3.87	0.00	0.21	0.05	0.16
Monod	3	-3.31	0.56	0.16	0.07	0.14
Quadratic	4	-3.15	0.72	0.15	0.06	0.24
Linear	3	-3.00	0.86	0.14	0.08	0.13
Exponential	3	-2.77	1.10	0.12	0.09	0.13
Null right slope	4	-2.65	1.22	0.12	0.07	0.22
Null	2	-2.19	1.68	0.09		
Null left slope	4	-0.09	3.77	0.00	0.22	0.13

**Sediment**	k	AICc	ΔAICc	w_i	p-value	R^2
Null	2	-12.90	0.00	1.00		
Exponential	3	-12.42	0.48	0	0.16	0.09
Linear	3	-12.36	0.54	0	0.17	0.09
Null right slope	4	-11.55	1.35	0	0.16	0.17
Quadratic	4	-9.81	3.09	0	0.33	0.10
Null left slope	4	-9.34	3.56	0	0.41	0.09
Power	NA					
Monod	NA					

**Nitrogen**	k	AICc	ΔAICc	w_i	p-value	R^2
Linear	3	-5.99	0.00	0.27	< 0.001	0.49
Exponential	3	-5.90	0.10	0.26	< 0.001	0.49
Power	4	-5.19	0.80	0.18	< 0.001	0.47
Null left slope	3	-3.69	2.30	0.09	< 0.001	0.50
Monod	3	-3.52	2.47	0.08	< 0.001	0.43
Quadratic	4	-3.09	2.90	0.06	< 0.001	0.49
Null right slope	2	-3.09	2.90	0.06	< 0.001	0.49
Null	4	7.41	13.40	< 0.01		

**Temperature**	k	AICc	ΔAICc	w_i	p-value	R^2
Null	2	-9.07	0.00	1.00		
Exponential	3	-6.43	2.64	0	0.88	< 0.01
Linear	3	-6.43	2.64	0	0.89	< 0.01
Power	3	-6.42	2.65	0	0.90	< 0.01
Monod	3	-6.41	2.65	0	0.92	< 0.01
Quadratic	4	-4.70	4.37	0	0.58	0.05
Null left slope	NA					
Null right slope	NA					

[a] Number of model parameters (including error), [b] function unable to fit the data
