# Supplementary material for: Ranking stressor impacts on periphyton structure and function with mesocosm experiments and environmental-change forecasts
Source: PLoS One. 2018 Sep 24;13(9):e0204510. doi: 10.1371/journal.pone.0204510 (PMC6152968; doi:10.1371/journal.pone.0204510)
Supplement: S4 Table — Models that did not explain sufficient variation in the periphyton response (i.e., AICc < null model AICc) were given a weight of 0 and not included in the model averaging (these models are highlighted in grey). Significance of the models (i.e. p-values) were determine by comparison to the null model with a likelihood ratio test (LRT). Model fits are reported as multiple R2 for linear models (linear, quadratic) and a quasi-R2 for non-linear models (squared correlation coefficient of predicted vs. observed Y). (PDF) [file pone.0204510.s005.pdf]

Table S4. Results of AIC multimodel inference and model weighting for stressor effects on phosphorus content. Models that did not explain sufficient variation in the periphyton response (i.e., AICc < null model AICc) were given a weight of 0 and not included in the model averaging (these models are highlighted in grey). Significance of the models (i.e. p-values) were determine by comparison to the null model with a likelihood ratio test (LRT). Model fits are reported as multiple R^2 for linear models (linear, quadratic) and a quasi-R^2 for non-linear models (squared correlation coefficient of predicted vs. observed Y).

**Extinction**	k[a]	AICc	ΔAICc	w_i	p-value	R^2
Null	2	-118.65	0.00	1.00		
Exponential	3	-117.58	1.07	0	0.24	0.06
Linear	3	-117.55	1.09	0	0.24	0.06
Null left slope	4	-115.23	3.42	0	0.40	0.08
Quadratic	4	-115.10	3.54	0	0.42	0.08
Null right slope	4	-114.63	4.02	0	0.52	0.06
Monod	3	-56.54	62.11	0	1.00	0.02
Power	3	-56.52	62.12	0	1.00	0.02

**Salt**	k	AICc	ΔAICc	w_i	p-value	R^2
Monod	3	-101.11	0.00	0.39	0.01	0.25
Power	3	-99.48	1.63	0.17	0.03	0.20
Null right slope	4	-99.05	2.06	0.14	0.03	0.28
Quadratic	4	-98.89	2.22	0.13	0.03	0.27
Linear	3	-97.37	3.74	0.06	0.09	0.13
Exponential	3	-97.13	3.98	0.05	0.10	0.12
Null	2	-96.76	4.35	0.04		
Null left slope	4	-94.15	6.96	0	0.28	0.11

**Phosphorus**	k	AICc	ΔAICc	w_i	p-value	R^2
Power	3	-98.05	0.00	0.45	< 0.001	0.72
Monod	3	-96.73	1.32	0.24	< 0.001	0.71
Quadratic	4	-95.47	2.58	0.13	< 0.001	0.73
Null right slope	4	-95.29	2.76	0.11	< 0.001	0.72
Linear	3	-93.73	4.32	0.05	< 0.001	0.67
Null left slope	4	-90.60	7.45	0.01	< 0.001	0.65
Exponential	3	-90.10	7.95	0.01	< 0.001	0.62
Null	2	-69.84	28.21	< 0.01		

**Sediment**	k	AICc	ΔAICc	w_i	p-value	R^2
Null	2	-115.76	0.00	1.00		
Exponential	3	-115.76	0.01	0	0.12	0.11
Linear	3	-115.74	0.02	0	0.12	0.11
Null left slope	4	-113.20	2.56	0	0.26	0.12
Quadratic	4	-112.82	2.94	0	0.31	0.11
Null right slope	4	-112.78	2.98	0	0.32	0.11
Power	3	-53.10	62.67	0	1.00	0.01
Monod	3	-52.99	62.77	0	1.00	< 0.01

**Nitrogen**	k	AICc	ΔAICc	w_i	p-value	R^2
Monod	3	-119.53	0.00	0.45	0.01	0.26
Power	3	-118.65	0.88	0.29	0.02	0.23
Linear	3	-117.27	2.27	0.15	0.03	0.19
Quadratic	4	-115.56	3.97	0.06	0.07	0.23
Null	2	-114.92	4.61	0.05		
Null left slope	4	-113.83	5.70	0	0.14	0.17
Null right slope	NA[b]					
Exponential	NA					

**Temperature**	k	AICc	ΔAICc	w_i	p-value	R^2
Null	2	-101.65	0.00	1.00		
Monod	3	-99.54	2.11	0	0.48	0.02
Power	3	-99.53	2.12	0	0.49	0.02
Linear	3	-99.53	2.12	0	0.49	0.02
Exponential	3	-99.52	2.13	0	0.49	0.02
Null right slope	4	-97.16	4.49	0	0.61	0.05
Quadratic	4	-96.77	4.89	0	0.73	0.03
Null left slope	4	-96.65	5.01	0	0.77	0.03

[a] Number of model parameters (including error), [b] function unable to fit the data
