# Supplementary material for: Ranking stressor impacts on periphyton structure and function with mesocosm experiments and environmental-change forecasts
Source: PLoS One. 2018 Sep 24;13(9):e0204510. doi: 10.1371/journal.pone.0204510 (PMC6152968; doi:10.1371/journal.pone.0204510)
Supplement: S1 Fig — Each panel represents a different stressor and the separate lines correspond to the six increasing levels of each stressor listed in Table 1 (i.e., Level 6 is highest treatment). Data are means ± SE of the two replicates with the exception of temperature, which had eleven unreplicated treatment levels. Ext. = extinction, N = nitrogen, Sed. = sediment, P = phosphorus, and Temp. = temperature. (PDF) [file pone.0204510.s006.pdf]

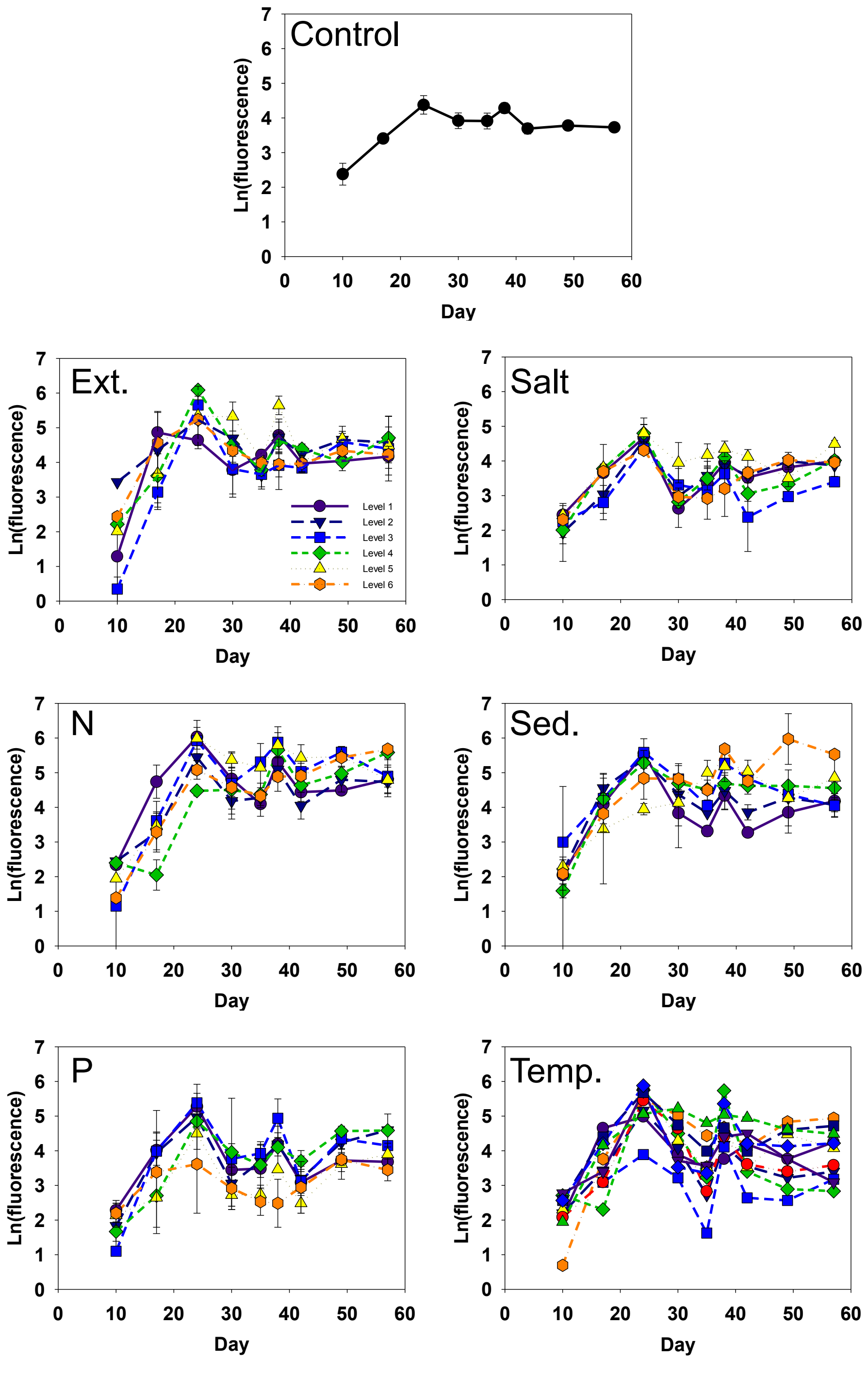

Figure S1. Periphyton growth expressed as fluorescence of chlorophyll *a* over time in the experiment. Each panel represents a different stressor and the separate lines correspond to the six increasing levels of each stressor listed in Table 1 (i.e., Level 6 is highest treatment). Data are means ± SE of the two replicates with the exception of temperature, which had eleven unreplicated treatment levels. Ext. = extinction, N = nitrogen, Sed. = sediment, P = phosphorus, and Temp. = temperature.
